# Lunch Provision, Consumption and Plate Waste in Early Years Settings in Sheffield

**DOI:** 10.1111/mcn.70132

**Published:** 2025-11-14

**Authors:** Claire J. Wall, Jo Pearce

**Affiliations:** ^1^ Sheffield Business School Sheffield Hallam University Sheffield UK

**Keywords:** child care, cross sectional analysis, food waste, lunch, nursery schools, nutritional intake, nutritional quality

## Abstract

Food provision in early years settings (EYS) presents an opportunity to support healthy eating amongst young children. This study aimed to record and nutritionally analyse setting lunches provided for, consumed and wasted by 3‐ to 4‐year‐old children attending EYS in Sheffield, England, including a comparison to packed lunches. Lunch choices were recorded for participating children, along with weights of foods served and any leftovers. A total of 142 setting lunches were recorded, eaten by 46 children attending four of eight recruited EYS. Lunches included vegetables (83.8%) more often than fruit (59.2%), and on average provided sufficient energy, carbohydrate, fibre, protein, vitamins A and C, calcium, iodine and zinc, but insufficient iron. Free sugars and saturated fat, but not sodium, were higher than recommended. Children left 22% of food served on their plate, and consumption of energy, carbohydrate, fibre, vitamin A, iron, iodine and zinc was lower than recommended. Food and nutrient contents were also compared to 185 packed lunches eaten by 67 children from eight settings. Setting lunches contained less food (median 288 g) than packed lunches (median 321 g, *p* < 0.001) and were more likely to meet guidelines for free sugars (*p* < 0.001), saturated fat (*p* < 0.001), vitamin A (*p* = 0.034), vitamin C (*p* < 0.001) and sodium (*p* < 0.001) but less frequently provided sufficient fibre (*p* = 0.025), calcium (*p* < 0.001), iron (*p* < 0.001) and zinc (*p* < 0.001). Setting lunches were more nutritionally balanced than packed lunches. However, to maximise EYS potential contribution to children's diets, settings must have access to support to both provide sufficiently nutrient‐dense meals and encourage children to eat them.

## Summary

- Setting lunches were generally well‐balanced in foods provided, but less commonly included fruit than vegetables.
- On average, setting lunches provided appropriate energy and carbohydrate, and sufficient protein, fibre, vitamin A, vitamin C, calcium, iodine and zinc, but insufficient iron. Free sugars and saturated fat, but not sodium, were above recommended maximums.
- Children left 22% of the food served to them. Consumption of energy, carbohydrate, fibre, vitamin A, iron, iodine and zinc was therefore lower than recommended.
- Packed lunches contained more food than setting lunches and were more energy‐dense, providing twice the free sugars, saturated fat and sodium than setting lunches.

## 1 Introduction

Ensuring that children receive a healthy diet during their first years of life is important to support growth and development, including promotion of healthy eating habits (Birch et al. 2007; Mameli et al. 2016). In comparison to dietary guidelines, diets of young children in the UK are high in free sugars and sodium, with protein consumption twice the Reference Nutrient Intake (Scientific Advisory Committee on Nutrition 2023). Meanwhile, diets are low in fibre and intakes of micronutrients such as iron, zinc and vitamin A may be inadequate amongst children from lower socioeconomic or minority ethnic groups (Scientific Advisory Committee on Nutrition 2023).

Almost all 3‐ and 4‐year‐old children in England (95%) are registered to receive their entitlement of 15 weekly hours of early education in term time and in 2024, there were 1.6 million registered childcare places in England across 54,700 early years settings (EYS) (Department for Education 2024a). The sector includes a diverse range of settings. Around half of childcare places (52%) are at private group settings (e.g. day nurseries), with a smaller proportion in school nurseries (22%), voluntary group settings (e.g. charity/community run pre‐schools; 14%) and with childminders (9%) (Department for Education 2024a).

Food provision in EYS provides an important avenue to support diet quality and health (Department for Education 2024b; World Health Organisation 2016; Yoong et al. 2023) and although settings are not required to provide food, where they do so, the Early Years Foundation Stage (EYFS) framework—the statutory framework that sets standards for early years providers—requires food to be ‘healthy, balanced and nutritious’ (Department for Education 2024c). National voluntary guidance has been produced previously to support settings to meet this requirement, including Eat Better, Start Better (EBSB) guidelines, which were revised in 2017 to provide voluntary food‐based guidelines for meals and snacks provided in settings (Action for Children 2017). More recently, EYFS nutrition guidance was introduced in September 2025, which EYS are required to ‘have regard to’ (Department for Education 2025a). The extent to which food guidance is followed by EYS appears mixed (Warren et al. 2023). Most of the previous research has been conducted in standalone nurseries, with little exploration of provision in school nurseries, where separate mandatory standards for school‐aged pupils are also in place (Wall and Pearce 2023).

It is important that food provision and the food environment within EYS meet children's nutritional needs and promote healthy eating behaviours to take full advantage of the opportunity that EYS provide. There is a lack of current evidence on food provision within EYS in England. Research conducted before the publication of updated EBSB guidance in 2017 suggested that lunches provided for children were low in energy, carbohydrate, iron and zinc, and high in sodium (Nicholas et al. 2013; Parker et al. 2011). Our previous research found that portion sizes were frequently not appropriate for 3‐ to 4‐year‐old children, and levels of energy, fat, carbohydrate, free sugars and sodium were higher than recommended for this age group (Pearce and Wall 2023; Wall and Pearce 2023), but this was conducted in school nurseries only, where children may have been served portions more appropriate for the older children also being catered for. Previous research has often focused on planned menus or food provision for children, rather than on what they choose and eat (Parker et al. 2011; Wall and Pearce 2023). Children are not eating all the food provided for them, and where data on plate wastage is available, this has been estimated at around 11%–24% of food served (Haroun et al. 2011; Nicholas et al. 2013). The highest proportion of wastage is typically for more nutrient‐dense foods such as vegetables, salad and fish. (Nicholas et al. 2013). It is, therefore, important to understand children's actual consumption to consider if their nutritional requirements are met, and to minimise waste from a cost and environmental perspective.

The aim of this study was to analyse the food, energy and nutrient content of lunches provided for, and eaten by, 3‐to 4‐year‐old children attending EYS, and the quantity of plate wastage by food group.

## 2 Methods

### 2.1 EYS Recruitment

Ethical approval was granted by Sheffield Hallam University research ethics review system (ID:ER61543155). Recruitment of EYS was described previously (Pearce and Wall 2025). In brief, EYS providing sessional or full day childcare on nondomestic premises (excluding holiday clubs) and primary/infant schools with nursery classes in Sheffield were identified in February 2024 from the Ofsted early years register and government school database respectively (Ofsted 2024; UK Government 2024). In total, 123 EYS and 79 schools were identified, contacted by email and invited to participate. EYS were eligible if nursery children were able to stay for lunch (provided by the setting and/or bringing packed lunches) and did not share a catering provider with any other recruited EYS. Purposive sampling was used to recruit EYS representing variety in setting type (to include school‐based nurseries, private nurseries and community nurseries/pre‐schools), area deprivation (to include EYS in areas of higher and lower deprivation measured using Indices of Deprivation (IMD)) and catering provider (with no two settings using the same catering provider).

Participating settings shared information sheets and consent forms with parents/carers of 3‐ to 4‐year‐olds in their nursery class (within schools) or pre‐school age group (in EYS). Parents provided informed consent for their children to participate, along with details of their child's age, sex and which days they attended the setting.

### 2.2 Data Collection

Data collection was conducted over five consecutive days in each EYS between April and July 2024. Before visiting each EYS, a researcher contacted the setting to discuss their lunchtime arrangements and request a copy of the lunch menu, and the recipes used to prepare the meals provided during the week of the visit.

During the daily study visits, a researcher asked kitchen staff to provide two portions of each menu option available for nursery children, in portion sizes they would serve to 3‐ and 4‐year old children. In schools, this usually included a main meal option (typically a meat or fish dish, served with starchy and/or vegetable accompaniments and dessert), a vegetarian option (typically a dish containing pulses, a meat alternative or cheese, served with starchy and/or vegetable accompaniments and dessert), a jacket potato option (a baked potato served with fillings such as baked beans, cheese or tuna, served with vegetable accompaniments and dessert) and a sandwich option (typically a filled sandwich, roll or wrap, served with vegetable accompaniments and dessert). In nurseries, this usually included a main meal option and a vegetarian option with accompaniments and dessert.

Each meal was separated into the different foods provided (e.g. roast chicken, roast potatoes, peas and carrots; pasta, bolognaise sauce and sweetcorn) and where possible, composite dishes were separated into individual ingredients (e.g. fillings separated from sandwiches, wraps and jacket potatoes). Each food item was weighed to the nearest 1 g using Salter kitchen scales, and each food item and weight recorded.

At lunchtime, the menu items chosen by participating children from the selection available at the counter or on the tables (depending on the individual setting) were recorded before they started to eat. Any second helpings were also recorded, and their trays or plates were collected when they had finished eating. Details of any food not eaten by the child, and weights of each leftover food item were then logged for each child. Settings were reimbursed for the cost of the lunches collected for weighing, and each received a £50 Amazon voucher to thank them for taking part.

### 2.3 Menu, Nutrient and Plate Waste Analysis

Details of the menu options chosen by each participating child, the portion weight of each item as served (based on weights of portions provided by kitchen staff), and the weight of any leftovers were entered into Excel and children's names were replaced by ID numbers. Where appropriate, edible portion weights were calculated using standard estimates (e.g. excluding the skin from watermelon slices, or the peel from bananas) (McCance and Widdowson 2014). The amount of each food item consumed by each child and the leftover weight for each item was then calculated by subtracting the leftover weights from the portion size as served. Any missing leftover weights (e.g., where a child's plate was not returned for weighing) were entered as the mean leftovers for each food chosen on that day.

Each food item was coded to record the food group (e.g. starchy food, dairy food, composite dish) and the specific food item (e.g. bread, raw fruit, yoghurt/fromage frais) based on food group categories included within EBSB guidance (Action for Children 2017).

Recipes for composite and cooked items were entered into Nutritics by the two researchers, both Registered Nutritionists with experience of using nutrient analysis software and evaluating school and nursery food provision (Nutritics 2025). Ingredients were entered using data from the UK food composition database (McCance and Widdowson 2014), with cooking methods applied to adjust for nutrient losses, and an overall weight change factor applied based on recipe type. Where details were provided of specific brands used, nutrient data was sourced from manufacturer or retailer websites and inputted, and the nearest match was used for any missing micronutrient data to avoid underestimation of micronutrient content. Queries regarding missing or unclear information were resolved by consensus and 10 percent of data entry and recipe analysis was checked to ensure accuracy and consistency in approach.

The energy and nutrient content of food provided for, and consumed by, each child, was estimated by entering the served weight and leftover weight of each food or recipe into a daily food log within Nutritics. Data were exported from Nutritics into Excel and uploaded into SPSS for analysis. The energy and nutrient contents of an average lunch were compared to the nutrient framework for lunches published within EBSB, which was calculated from dietary reference values for children aged 1–4 years (Action for Children 2017). Energy and nutrient contents of setting lunches were also compared to those for packed lunches collected during the same visits. Full details of the nutritional analysis of the packed lunches have been published separately (Pearce and Wall 2025), but followed a similar approach to analysis of setting lunches, with lunchbox contents and portion weights for each item recorded before lunch was eaten, and leftovers collected and weighed after lunch. Details of the items provided, and the provided and leftover weights were then used to create daily food logs for each lunch in Nutritics, before being exported to Excel and then SPSS.

Each individual setting lunch was also coded to indicate the presence or absence of specific foods and food groups as recommended to provide (e.g. fruit, vegetables, wholegrain starchy foods) or limit/avoid (e.g. meat products, cakes, confectionery) within EBSB guidance and/or EYFS nutrition guidance (Action for Children 2017; Department for Education 2025a). Food item and food group coding was used to calculate the average plate waste (in grams and as a proportion of the served portion) for each food group or food item.

### 2.4 Statistical Analysis

Data on energy and nutrient content of lunches were not normally distributed and are presented as medians, with mean values also stated for comparison with previous research and nutrient frameworks. Quantity of food provided by lunch type was compared using a Mann–Whitney U test. The nutrient content of setting lunches was compared with packed lunches observed as part of the same study and reported separately using an analysis of covariance (ANCOVA) test (Pearce and Wall 2025). This was conducted at the individual lunch level, adjusting for children's age, sex, ID number and setting attended to account for food provision being similar for children attending the same setting on the same day, but packed lunches for individual children being similar across the week. A chi‐squared test was used to determine whether the proportion of setting lunches that met the nutrient framework differed significantly between setting lunches and packed lunches. A value of *p* < 0.05 was used to describe statistical significance in all cases.

### 2.5 Ethics statement

This study was conducted according to the guidelines laid down in the Declaration of Helsinki and all procedures involving research study participants were approved by the Sheffield Hallam University research ethics review system (ID:ER61543155). Written informed consent was obtained from all subjects. The lead author affirms that this manuscript is an honest, accurate, and transparent account of the study being reported. The reporting of this work is compliant with STROBE guidelines. The lead author affirms that no important aspects of the study have been omitted and that any discrepancies from the study as planned have been explained.

## 3 Results

### 3.1 Participant and Setting Characteristics

Two school‐based nurseries, four private nurseries and two community nurseries/pre‐schools were recruited to take part in the study. Setting lunches were available for children in five of the eight recruited EYS, but consent was only provided by parents for children consuming a setting lunch at four of these five settings (two school‐based nurseries, one community nursery and one private nursery). Three settings were situated in areas with higher deprivation (Indices of Multiple Deprivation (IMD) deciles 1–5) whilst one was in an area of lower deprivation (IMD 6–10) (Table 1). A weighed lunchtime intake was recorded for 46 children eating a total of 144 setting lunches across the four included settings. Two lunches were excluded from the study, because on that lunch occasion, the child also ate lunch items brought from home. A further 185 packed lunches eaten by 67 children were also recorded from the eight EYS that took part in the study. Detailed findings from the packed lunches are reported separately (Pearce and Wall 2025).

**Table 1:** Participant and setting characteristics.

Participant characteristics	*n* (%)
Boys	20 (43%)
Girls	26 (57%)
Aged 3 years	15 (33%)
Aged 4 years	31 (67%)
Total number of setting lunches observed	142
Mean no. of setting lunches per child (standard deviation)	3.1 (1.4)
Median no. of setting lunches per child (interquartile range)	3.0 (1.5–4.5)
Range (number) of setting‐provided lunches per child	1–5

Setting characteristics	*n* (%)
Settings	8 (100%)
Settings where setting lunches were observed	4 (50%)
Setting type (where setting lunches observed):	
School‐based nursery	2 (50%)
Private day nursery	1 (25%)
Community nursery	1 (25%)
Area deprivation:	
Higher (IMD deciles 1–5)	3 (75%)
Lower (IMD deciles 6–10)	1 (25%)

Abbreviation: IMD, indices of multiple deprivation.

Slightly more girls (57%) and more children were aged 4 years (67%) ate setting lunches during the study. The mean number of setting lunches per child was 3.1 (median 3.0) with between one and five lunches recorded for each child (Table 1).

### 3.2 Food Items Provided

**Figure 1:**
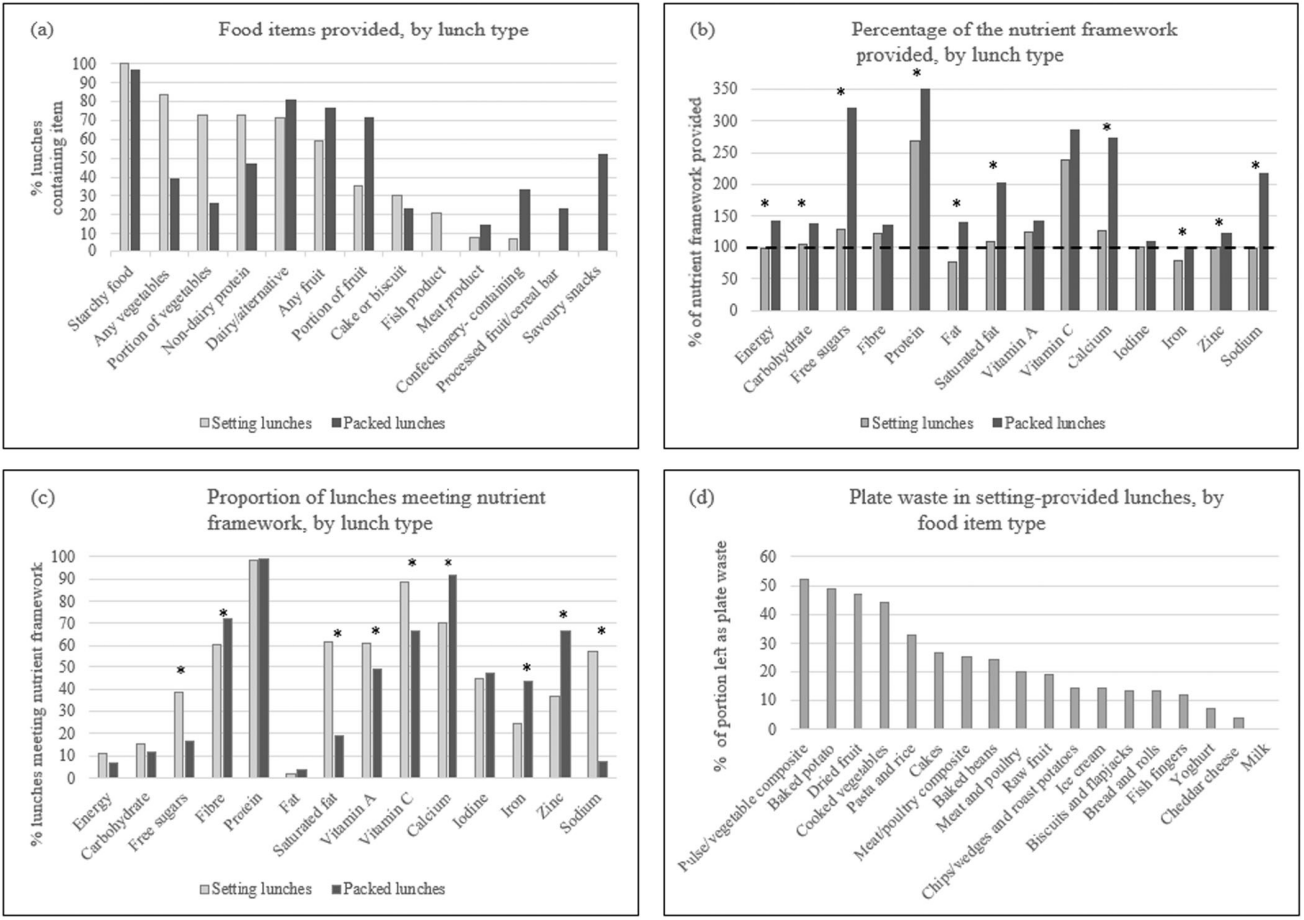
Food types provided and comparison with nutrient frameworks by lunch type, and plate waste by food group in setting‐provided lunches. (a) Food items provided, by lunch type. (b) Percentage of the nutrient framework provided, by lunch type. (c) Proportion of lunches meeting nutrient framework, by lunch type. (d) Plate waste in setting‐provided lunches, by food item type. *Denotes a statistical difference between lunch types, *p* < 0.05 in Figures 1b,c.

All provided setting lunches contained a starchy food (100%) (Table 2), and most lunches provided vegetables (83.8%), a portion of vegetables (≥ 40 g) (72.5%), a non‐dairy source of protein (72.5%) and a dairy food (or plant‐based alternative to dairy) (71.1%). Fruit was included in 59.2% of lunches but only 35.2% of lunches contained a portion of fruit (≥ 40 g) and few lunches contained a wholegrain starchy food (3.5%). Lunches also often included fried starchy foods (40.1%), fish products (21.1%) and meat products (7.7%). Cakes and biscuits were provided in 30.3% of lunches and confectionery‐containing items in 7.0% of lunches, but no savoury snacks, processed fruit bars or cereal bars were observed. A comparison was made between setting and packed lunches (Figure 1a). Setting lunches more frequently contained vegetables, a portion of vegetables, a non‐dairy source of protein, cakes and biscuits and fish products, but less frequently contained dairy/alternatives, fruit, a portion of fruit, meat products, confectionery, processed fruit and cereal bars and savoury snacks when compared to packed lunches.

**Table 2:** Percentage of setting‐provided lunches (*n* = 142) containing specific food items.

Food item or group	Percentage of lunches containing food item
Fruit	59.2
A portion of fruit (≥ 40 g)	35.2
Vegetables	83.8
A portion of vegetables (≥ 40 g)	72.5
Starchy food	100.0
Wholegrain starchy food	3.5
Fried starchy food (e.g. chips)	40.1
Non‐dairy protein	72.5
Dairy or alternative	71.1
Meat product (e.g. sausage roll)	7.7
Fish product (e.g. fish finger)	21.1
Cake or biscuit	30.3
Item containing chocolate or sugar confectionery	7.0
Processed fruit or cereal bar	0.0
Savoury snacks (e.g. crisps)	0.0

### 3.3 Nutrient Content of Setting Lunches as Provided and Consumed

On average, setting lunches met lunchtime nutrient guidelines for energy, carbohydrate, fibre, protein, vitamin A, vitamin C, calcium, iodine, zinc and sodium (Table 3). Average provision of free sugars and saturated fat were higher than recommended, whilst provision of total fat and iron were lower than recommended (Table 3). Setting lunches provided over twice the recommended amount of protein (Figure 1b).

**Table 3:** Nutrient content of setting‐provided lunches as provided to, and consumed by, children.

Nutrient	Nutrient framework for lunches^a^	As provided (*n* = 142)				As consumed (*n* = 142)			
		Mean	Standard deviation	Median	Interquartile range	Mean	Standard deviation	Median	Interquartile range
Energy (kJ)	~1542 (1465–1620)	1506	583	1426	783–2069	1147	557	1077	664–1490
Energy (kcal)	~369 (351–387)	358	139	337	260–415	272	132	256	158–355
Carbohydrate (g)	~49.2 (46.7–51.7)	50.8	18.5	51.0	40.9–61.1	38.1	17.9	36.4	25.3–47.6
Free sugars (g)	≤ 4.9	6.3	6.1	5.6	1.4–9.8	4.6	5.2	3.5	0.3–8.7
Fibre (g)	≥ 4.5	5.5	2.2	5.3	4.1–6.5	3.9	2.1	3.5	1.9–5.1
Protein (g)	≥ 5.1	13.7	4.3	13.5	10.9–16.1	10.6	4.7	10.1	6.8–13.5
Fat (g)	~14.4 (13.7–15.1)	11.0	6.9	9.8	4.9–14.8	8.6	6.0	6.9	2.2–11.6
Saturated fat (g)^b^	≤ 4.1	4.4	3.0	3.3	1.3–5.3	3.4	2.7	2.8	0.8–4.9
Vitamin A (µg)	≥ 136	170	134	151	111–192	107	97	93	49–138
Vitamin C (mg)	≥ 9	21.4	14.0	18.5	11.4–25.7	15.3	13.2	11.4	5.3–17.6
Calcium (mg)	≥ 120	152	74	139	93–186	124	71	120	79–161
Iodine (µg)^b^	≥ 26	26	20	23	10–37	22	19	18	5.5–30.5
Iron (mg)	≥ 2.3	1.8	0.7	1.6	1.2–2.0	1.3	0.7	1.3	0.8–1.8
Zinc (mg)	≥ 1.7	1.7	0.7	1.5	1.2–1.9	1.2	0.7	1.1	0.8–1.5
Sodium (mg)	≤ 300	295	188	274	150–398	242	181	208	83–333

^a^ The nutrient framework for lunches represents nutrient‐based standards for lunches for children aged one to four years attending early years settings (Action for Children 2017). Where an approximate value is given (for energy, carbohydrate and fat), a range (±5%) has been calculated to support a comparison with the standard.

^b^ Saturated fat and iodine are not included in the nutrient framework but are included here due to concerns about intakes in young children. These have been calculated from UK dietary reference values (saturated fat to provide ≤ 10% energy; iodine to provide ≥ 30% of the RNI for children aged 3–4 years in line with the method of calculation for other micronutrients within the nutrient framework).

Average nutrient consumption was in line with guidelines for free sugars, protein, saturated fat, vitamin C, calcium and sodium. Children consumed less than the recommended amounts of energy, carbohydrate, fibre, total fat, vitamin A, iodine, iron and zinc (Table 3).

### 3.4 Comparison of Setting Lunches and Packed Lunches (as Provided)

The average energy and nutrient content of setting lunches (142 lunches from four EYS) was compared to packed lunches (185 lunches from eight EYS), as provided for children attending the eight EYS that took part in the study (Table 4, Figure 1b) (Pearce and Wall 2025). On average, packed lunches contained more food than setting lunches (median 321 g vs. 288 g, *p* < 0.001) and were more energy‐dense (providing median 1.6 kcal/g vs. 1.2 kcal/g, *p* < 0.001) (data not shown), containing significantly more energy overall (median 507 kcal vs. 337 kcal, *p* < 0.001). On average, packed lunches also contained significantly more carbohydrate (median 62.0 g, vs. 51.0 g, *p* < 0.001), protein (median 17.6 g vs. 13.5 g, *p* < 0.001), total fat (median 20.1 g vs. 9.8 g, *p* < 0.001), calcium (median 304 mg, vs. 139 mg, *p* < 0.001), iron (median 2.2 mg, vs. 1.6 mg, *p* < 0.001) and zinc (median 2.1 mg vs. 1.5 mg, *p* < 0.001), but also significantly more free sugars (median 11.9 g vs. 5.6 g, *p* < 0.001) saturated fat (median 8.1 g vs. 3.3 g, *p* < 0.001) and sodium (median 616 mg vs. 274 mg, *p* < 0.001) when compared to setting lunches.

**Table 4:** Energy and nutrient content of setting‐provided lunches in comparison to packed lunches.

Nutrient	Nutrient framework for lunches^a^		All lunches (*n* = 327)				Setting‐provided lunches (*n* = 142)				Packed lunches (*n* = 185)				*p* value^c^
			Mean	SD	Median	IQR	Mean	SD	Median	IQR	Mean	SD	Median	IQR	
Energy (kJ)		~1542 (1465–1620)	1901	717	1849	1349–2349	1506	583	1426	783–2069	2203	663	2138	1734–2542	< 0.001
Energy (kcal)		~369 (351–387)	452	171	440	321–559	358	139	337	260–415	525	158	507	412–603	< 0.001
Carbohydrate (g)		~49.2 (46.7–51.7)	60.3	24.0	54.6	39.8–69.4	50.8	18.5	51.0	40.9–61.1	67.6	25.2	62.0	43.9–80.1	< 0.001
Free sugars (g)	≤ 4.9		11.6	11.6	8.0	2.0–14.0	6.3	6.1	5.6	1.4–9.8	15.7	13.1	11.9	2.3–21.5	< 0.001
Fibre (g)	≥ 4.5		5.8	2.4	5.4	3.8–7.1	5.5	2.2	5.3	4.1–6.5	6.1	2.5	5.8	4.1–7.5	0.653
Protein (g)	≥ 5.1		16.1	6.2	15.6	11.8–19.4	13.7	4.3	13.5	10.9–16.1	18.0	6.8	17.6	14.1–21.1	< 0.001
Fat (g)	~14.4		16.1	9.1	15.8	9.1–22.6	11.0	6.9	9.8	4.9–14.8	20.1	8.6	20.1	14.5–25.8	< 0.001
Saturated fat (g)^b^	≤ 4.1		6.6	4.4	5.9	2.7–9.1	4.4	3.0	3.3	1.3–5.3	8.3	4.5	8.1	4.5–11.7	< 0.001
Vitamin A (µg)	≥ 136		183	207	149	90–208	170	134	151	111–192	193	249	135	60–210	0.229
Vitamin C (mg)	≥ 9		23.8	20.3	19.8	8.2–31.4	21.4	14.0	18.5	11.4–25.7	25.7	23.9	22.0	6.1–38.0	0.637
Calcium (mg)	≥ 120		252	160	207	103–311	152	74	139	93–186	328	166	304	185–423	< 0.001
Iodine (µg)^b^	≥ 26		27.2	20.4	24.0	11.2–36.8	26	20	23	10–37	28	21	25	14–37	0.101
Iron (mg)	≥ 2.3		2.1	0.9	2.0	1.5–2.5	1.8	0.7	1.6	1.2–2.0	2.3	0.9	2.2	1.6–2.8	< 0.001
Zinc (mg)	≥ 1.7		1.9	0.8	1.8	1.2–2.4	1.7	0.7	1.5	1.2–1.9	2.1	0.8	2.1	1.5–2.7	< 0.001
Sodium (mg)	≤ 300		496	318	453	258–649	295	188	274	150–398	651	311	616	421–812	< 0.001

Abbreviations: IQR, interquartile range; SD, standard deviation.

^a^ The nutrient framework for lunches represents nutrient‐based standards for lunches for children aged one to four years attending early years settings (Action for Children 2017). Where an approximate value is given (for energy, carbohydrate and fat), a range (±5%) has been calculated to support a comparison with the standard.

^b^ Saturated fat and iodine are not included in the nutrient framework but are included here due to concerns about intakes in young children. These have been calculated from UK dietary reference values (saturated fat to provide ≤ 10% energy; iodine to provide ≥ 30% of the RNI for children aged 3–4 years in line with the method of calculation for other micronutrients within the nutrient framework).

^c^ p values from analysis of covariance (ANCOVA), comparing the energy/nutrient content of setting‐provided lunches with packed lunches brought by children from home, adjusting for child's age, sex and ID and the setting ID.

A significantly higher proportion of setting lunches met the nutrient guidelines for free sugars (38.7% vs. 16.8%, *p* < 0.001), saturated fat (62.0% vs. 18.9%, *p* < 0.001), vitamin A (61.3% vs. 49.2%, *p* = 0.034), vitamin C (88.7% vs. 66.5%, *p* < 0.001) and sodium (57.7% vs. 7.6%, *p* < 0.001) when compared to packed lunches. A higher proportion of packed lunches met nutrient recommendations for fibre (72.4% vs. 60.6%, *p* = 0.025), calcium (91.4% vs. 70.4%, *p* < 0.001), iron (43.2% vs. 24.6%, *p* < 0.001) and zinc (66.5% vs. 36.6%, *p* < 0.001). No differences were observed for energy (*p* = 0.162), carbohydrate (*p* = 0.322), total fat (*p* = 0.523), protein (1.000) or iodine (*p* = 0.655) (Figure 1c).

### 3.5 Portion Sizes of Setting Lunches

Average portions of baked potatoes and milk were smaller than typical portion sizes stated in EBSB guidance (Table 5) (Action for Children 2017). Portions of baked beans, bread, cheddar cheese, raw fruit and yoghurt were slightly larger than recommended, whilst portions of cooked vegetables, meat and poultry, meat/poultry composite dishes, pasta and rice and dried fruit were within range.

**Table 5:** Portion sizes and plate waste by food group in setting provided lunches (*n* = 142).

Food item	Number of lunches containing food item	Mean portion size (g (SD))	Typical recommended portion sizes where available (g unless otherwise stated)^a^	Mean plate waste (g (SD))	Mean % consumed by children
Baked beans	10	72 (19)	55	16 (14)	76
Biscuits and flapjacks	21	24 (25)	—	7 (15)	87
Baked potato	24	66 (78)	80–100	23 (41)	51
Bread and rolls	32	35 (17)	20–30	4 (9)	87
Cakes	24	39 (9)	—	12 (17)	73
Chips, wedges and roast potatoes	57	79 (24)	—	11 (19)	86
Cooked vegetables	110	40 (19)	40	16 (14)	56
Dried fruit	14	18 (0)	15–30	10 (10)	53
Fish fingers	39	36 (20)	—	5 (14)	88
Cheddar cheese	17	24 (12)	15–20	1 (3)	96
Ice cream	11	36 (2)	—	5 (12)	86
Meat or poultry composite dish	16	115 (48)	90–120	21 (23)	75
Milk	40	80 (31)	100–150 ml	0 (0)	100
Pasta and rice	30	89 (28)	80–100	27 (17)	67
Meat and poultry	13	39 (16)	30–40	8 (10)	80
Pulse/vegetable composite dishes	47	162 (57)	90–120	78 (47)	48
Raw fruit	67	48 (24)	40	11 (19)	81
Yoghurt	11	77 (23)	50–75	6 (6)	93

Abbreviations: g, grams; SD, standard deviation.

^a^ Typical portion sizes as stated in Eat Better, Start Better guidance (Action for Children 2017).

### 3.6 Plate Waste for Setting Lunches

Overall, children left over a fifth (median 22.0%, mean 24.8%) of the food served on their plates. The foods with the largest proportion wasted (left on the plate) were pulse/vegetable composite dishes (mean 52%), baked potatoes (49%), dried fruit (47%), cooked vegetables (44%), pasta and rice (33%) and cakes (27%) (Table 5). Foods with the least plate waste were milk (0%), cheddar cheese (4%), yoghurt (7%), fish fingers (12%), bread/bread rolls (13%), chips, wedges and roast potatoes (14%) and ice cream (14%) (Figure 1d).

## 4 Discussion

The aim of this study was to record the types of food provided to 3‐ and 4‐year‐old children attending EYS, estimate the energy and nutrient content of setting lunches as provided and eaten by children and the quantity of plate wastage by food group, as well as comparing setting lunches to packed lunches brought from home.

Setting lunches usually provided the foods recommended by EBSB guidance, including starchy foods, vegetables, fruit, non‐dairy sources of protein and dairy/alternative foods. This is very encouraging and in line with previous research. Fruit (provided within 59.2% of lunches in this study) and vegetables (within 83.8% of lunches) were more frequently provided than in the pre‐school food survey (research conducted in 2012 detailing food provision across 57 EYS in England), where 36% and 54% of lunches contained discrete portions of fruit and vegetables respectively (Nicholas et al. 2013). In a recent analysis of key stage 1 children (aged 4–7 years), 70.2% and 92.4% of children consumed fruit and vegetables as part of their lunch (Haney et al. 2023).

Although setting lunches also contained foods that are recommended to limit or avoid (e.g., fried starchy foods, cakes, biscuits, confectionery, fish products and meat products), provision of these foods was consistent with previous research when settings were visited every day of the week, with many settings, particularly those in schools, offering fish fingers and chips as a popular fish option on Friday, a day where schools are traditionally expected to serve fish in place of meat (Pearce and Wall 2023; Wall and Pearce 2023).

Setting lunches met most nutrient guidelines (for energy, carbohydrate, fibre, protein, vitamin A, vitamin C, calcium, iodine, zinc and sodium) but provision of free sugars and saturated fat were higher than recommended and provision of total fat and iron were lower than recommended. The energy content of lunches (358 kcal) was within the range stated in EBSB guidance (351–387 kcal) and was lower than our previous study carried out in nine school‐based nurseries (450 kcal) but higher than meals (as plated for children) in the pre‐school food survey (310 kcal) (Nicholas et al. 2013; Wall and Pearce 2023). In our previous study, we suggested the higher‐than‐recommended energy content was due to provision being planned for the older children in schools, for whom standards are mandatory and not voluntary (Wall and Pearce 2023). Half the EYS included in the current study were schools, suggesting that the energy content of meals provided in the private or community settings could be lower than recommended. Wide variation in the energy content of setting lunches observed in the current study also explains why a relatively small proportion of individual lunches met the nutrient framework for energy, while average provision was appropriate. Provision of free sugars (mean 6.3 g) and saturated fat (mean 4.4 g) were higher than recommended in this study, an issue which persists in setting meals for children of all ages (Haney et al. 2023; Wall and Pearce 2023). Content was, however, lower than in both the pre‐school food survey (7.9 g of NMES and 5.0 g saturated fat) and our previous study in school‐based nurseries (10.5 g free sugars and 11 g saturated fat), indicating either smaller portion sizes of foods such as cakes and biscuits, or less frequent provision in other types of EYS (Nicholas et al. 2013; Wall and Pearce 2023). Overall, however, findings from this study suggest provision of cakes, biscuits and other desserts were still slightly higher than the once a week suggested by EBSB guidance or complete avoidance stated within the new EYFS nutrition guidance (Department for Education 2025a).

The mean protein content of setting lunches (13.7 g) was also high and whilst protein supports growth and development, and provision was consistent with previous studies in young children (Lennox et al. 2013; Nicholas et al. 2013; Office for Health Improvement and Disparities 2025; Pearce and Wall 2025; Wall and Pearce 2023), the association between high protein intake and higher body mass index in children has been recently highlighted by the Scientific Advisory Committee on Nutrition (Scientific Advisory Committee on Nutrition 2023). Reducing provision of protein may, however, impact on provision of other nutrients contained within protein‐rich foods such as iron and zinc in meat, and fibre in beans and pulses. The iron content of meals was lower than recommended and reducing portion sizes or frequency of meat may reduce iron content further.

Whilst provision of most nutrients in setting lunches was favourable, children left 22% of the food served and so consumption of some key nutrients (energy, carbohydrate, fibre, total fat, vitamin A, iodine, iron and zinc) was lower than recommended. Compared to the pre‐school food survey, however, intake was remarkably similar for both energy (272 and 267 kcal in this study and the pre‐school study respectively) and macronutrients (Nicholas et al. 2013). Intakes of micronutrients were also similar, for example, 15.3 mg vitamin C, 124 mg calcium and 1.3 g iron, compared to 15.8 mg vitamin C, 125 mg calcium and 1.2 g iron in the pre‐school food survey, despite more pronounced differences in provision (Nicholas et al. 2013). Intake from setting lunches was also lower than from packed lunches (mean 416 kcal), where children also left around a fifth (median 16.1%, mean 20.6%) of their lunch (Pearce and Wall 2025). There is evidence that young children exhibit satiety responsiveness and stop eating when they are full, rather than consuming all the food provided (Birch and Fisher 1998), and rejection of new or unfamiliar foods and fussy eating are also relatively common in this age group (Dovey et al. 2008). This highlights the challenge for EYS of providing sufficiently energy and nutrient‐dense foods, without providing excess free sugars and saturated fat, and then encouraging children to eat them.

Portion sizes of setting lunches were broadly aligned with typical portion sizes from EBSB guidance (Action for Children 2017). As the portion sizes stated within this guidance are for children aged 1–4 years, where they are stated as a range (e.g. 80–100 g for starchy foods), it would be appropriate for portion sizes provided for children aged 3–4 years (as in this study) to be at the upper end of the range. This appeared to be the case for many commonly provided foods (e.g. meat composite dishes, cheese, meat and poultry, raw fruit, yoghurt) which were towards the top, or slightly above, the stated range. Portion sizes varied within some food groups, with portions of pasta and rice within range, but portions of baked potatoes much smaller. Apart from baked beans, rice and chips, portion sizes were consistently smaller than those recently observed in 10 school nurseries in Sheffield (Pearce and Wall 2023). This may, again, reflect the inclusion of a wider range of settings (private and community nurseries in addition to school nurseries) in the current study. Portions of cakes and biscuits were previously found in school‐based nurseries to be large (39–58 g) (Pearce and Wall 2023), but were smaller (24–39 g) in the current study, which will have contributed to lower mean free sugars content of lunches seen in this study (6.3 g) compared with only school‐based nurseries (10.5 g) (Wall and Pearce 2023).

Although portion sizes were broadly appropriate, children left over a fifth (22%) of the food provided in setting lunches on their plate. Plate wastage was similar between setting lunches and packed lunches (where 16% of food provided was left), and in line with plate waste observed for primary school lunches in 2009 (24%), but higher than the 2012 pre‐school food survey (11%) where portion sizes and leftovers were estimated by setting staff using a food atlas (Haroun et al. 2011; Nicholas et al. 2013). Setting lunch plate waste for vegetables, vegetable dishes and salad were amongst the highest across these studies, highlighting that despite full portions being provided in EYS and schools, they are not always eaten (Haroun et al. 2011; Nicholas et al. 2013). Despite portion sizes of starchy foods being appropriate (pasta and rice) or low (baked potatoes), plate waste for these foods was also amongst the highest across the different foods observed in this study. This meant that although food provision met the nutrient framework for energy, carbohydrate and fibre, children's intake of these nutrients was low. In addition to nutritional considerations, high plate wastage also has economic and environmental consequences related to the purchasing and preparation of foods that are not eaten (Biasini et al. 2024).

Recent government statutory guidance has clarified that families must be able to access funded childcare free of charge, and can, therefore, choose to provide a packed lunch instead of paying for setting meals (Department for Education 2025d). This study is consistent with other research in EYS and schools in finding that setting lunches are of higher nutritional quality than packed lunches (Haney et al. 2023; Nicholas et al. 2013; Pearce et al. 2011). Vegetables and non‐dairy protein were more frequently provided in setting lunches, and items recommended or required to be limited or avoided in EYS and schools (e.g. meat products, confectionery‐containing items, processed fruit bars, savoury snacks) were less commonly provided than in packed lunches. Setting lunches were lower in free sugars, saturated fat and sodium than packed lunches, and although recommended limits for these nutrients were not met in all setting lunches, this was significantly more likely than for packed lunches. Packed lunches did have some advantages – they more commonly included a dairy food and a full portion of fruit and were slightly less likely to include cakes and biscuits. Levels of several micronutrients were more likely to meet the nutrient framework in packed lunches, including calcium, iron and zinc, which are of particular importance for this age group (Scientific Advisory Committee on Nutrition 2023).

Current expansion of funded childcare hours (Department for Education 2023), and an additional 6,000 school nursery places (Department for Education 2025c), will increase the number of children attending EYS and potentially eating setting lunches. Changes to free school meal entitlement will also increase the proportion of children attending school nurseries that are eligible for free lunches (Department for Education 2025b). The findings from this study highlight areas where setting lunches (and packed lunches) provided for children can be further improved to maximise the opportunity that this provides. New EYFS nutrition guidance came into effect in September 2025 which will strengthen guidance around limiting foods high in free sugars, saturated fat and sodium (e.g. by stating that cakes and biscuits should no longer be provided) (Department for Education 2025a). As consumption of energy and several key nutrients were shown to be low in this study, it will be important to ensure that lunches provide sufficient energy, carbohydrate, fibre and micronutrients alongside the continued focus on reducing sugar, saturated fat and salt. Updates to school food standards are also underway (Department for Education 2025b), and schools with nurseries may need particular support to ensure they can meet the EYFS nutrition guidance for nursery children alongside the standards for older children.

This study is the first in the UK to collect weighed intake data on the food provided and consumed but the study did have several limitations. It was a small study, with setting lunches observed in four of eight recruited EYS (two of which were school‐based), was conducted in one geographical area and may not be representative of food provision locally or nationally. Families were asked to provide consent for their children to take part in the study but not all of them consented and EYS were only visited for 5 days. This study does, however, make an important contribution to a very limited evidence base at a time of significant policy change.

## 5 Conclusion

Setting lunches were generally well balanced in the types of foods provided, but provision of fruit was relatively low, and foods that EYFS guidance for EYS now recommends being avoided (e.g. cakes and biscuits) were commonly provided. Setting lunches provided appropriate amounts of most nutrients, but were high in free sugars and saturated fat, and low in iron. High levels of plate waste meant that intakes of energy and several key nutrients was low. Setting lunches contained less food than packed lunches, and were lower in free sugars, saturated fat and sodium, but also provided less calcium, iron and zinc. With extensions to funded childcare and entitlement to free school meals likely to increase the number of children eating in EYS, continued support to improve both setting and packed lunches is required.

## Author Contributions

Claire J. Wall and Jo Pearce jointly designed the research, collected data, analysed the data, and wrote the paper. Both authors have read and approved the final manuscript.

## Conflicts of Interest

Claire J. Wall was involved in the development of the voluntary food and drink guidelines for early years settings in England (Eat Better, Start Better) and has provided consultancy services to Action for Children. JP has no conflicts of interest to declare.

## Data Availability

The data that support the findings of this study are available from the corresponding author upon reasonable request.

## References

[mcn70132-bib-0001] Action for Children . 2017. *Eat Better, Start Better. Voluntary Food and Drink Guidelines for Early Years Settings in England*. https://foundationyears.org.uk/eat-better-start-better/.

[mcn70132-bib-0002] Biasini, B. , M. Donati , A. Rosi , et al. 2024. “Nutritional, Environmental and Economic Implications of Children Plate Waste at School: A Comparison Between Two Italian Case Studies.” Public Health Nutrition 27: e143. 10.1017/S136898002400034X. PMC11374544 38361449

[mcn70132-bib-0003] Birch, L. , J. S. Savage , and A. Ventura . 2007. “Influences on the Development of Children's Eating Behaviours: From Infancy to Adolescence.” Canadian Journal of Dietetic Practice and Research: A Publication of Dietitians of Canada = Revue Canadienne de la Pratique et de la Recherche en Dietetique: Une Publication des Dietetistes du Canada 68, no. 1: 1. https://www.ncbi.nlm.nih.gov/pubmed/19430591. PMC2678872 19430591

[mcn70132-bib-0004] Birch, L. L. , and J. O. Fisher . 1998. “Development of Eating Behaviors Among Children and Adolescents.” Pediatrics 101, no. 2: 539–549. 10.1542/peds.101.S2.539. 12224660

[mcn70132-bib-0005] Department for Education . 2023, 01 June. Free Childcare: How We Are Tackling the Cost of Childcare. Department for Education. https://educationhub.blog.gov.uk/2023/06/01/free-childcare-how-we-tackling-the-cost-of-childcare/.

[mcn70132-bib-0006] Department for Education . 2024a. Childcare and Early Years Provider Survey. 2024 Reporting Year. Department for Education. https://explore-education-statistics.service.gov.uk/find-statistics/childcare-and-early-years-provider-survey/2024.

[mcn70132-bib-0007] Department for Education . 2024b. Education Provision: Children Under 5 Years of Age. Department for Education. https://explore-education-statistics.service.gov.uk/find-statistics/education-provision-children-under-5.

[mcn70132-bib-0009] Department for Education . 2024c. Statutory Framework for the Early Years Foundation Stage. Department for Education. https://www.gov.uk/government/publications/early-years-foundation-stage-framework--2.

[mcn70132-bib-0010] Department for Education . 2025a, 17 April. Early Years Foundation Stage nutrition guidance. Department for Education. https://www.gov.uk/government/publications/early-years-foundation-stage-nutrition.

[mcn70132-bib-0011] Department for Education . 2025b, 4 June. Over Half a Million More Children to get Free School Meals. Department for Education. https://www.gov.uk/government/news/over-half-a-million-more-children-to-get-free-school-meals.

[mcn70132-bib-0012] Department for Education . 2025c, 2 April. Parents to Save Thousands Through School‐Based Nursery Places. Department for Education. https://www.gov.uk/government/news/parents-to-save-thousands-through-school-based-nursery-places.

[mcn70132-bib-0013] Department for Education . 2025d, 21 February. Statutory Guidance: Early Education and Childcare. Department for Education. https://www.gov.uk/government/publications/early-education-and-childcare--2.

[mcn70132-bib-0014] Dovey, T. M. , P. A. Staples , E. L. Gibson , and J. C. G. Halford . 2008. “Food Neophobia and ‘Picky/Fussy’ Eating in Children: A Review.” Appetite 50, no. 2–3: 181–193. 10.1016/j.appet.2007.09.009. 17997196

[mcn70132-bib-0015] Haney, E. , J. C. Parnham , K. Chang , et al. 2023. “Dietary Quality of School Meals and Packed Lunches: A National Study of Primary and Secondary Schoolchildren in the UK.” Public Health Nutrition 26, no. 2: 425–436. 10.1017/S1368980022001355. 35641314 PMC13076075

[mcn70132-bib-0016] Haroun, D. , C. Harper , L. Wood , and M. Nelson . 2011. “The Impact of the Food‐Based and Nutrient‐Based Standards on Lunchtime Food and Drink Provision and Consumption in Primary Schools in England.” Public Health Nutrition 14, no. 2: 209–218. 10.1017/S1368980010002132. 20701821

[mcn70132-bib-0017] Lennox, A. , J. Sommerville , K. Ong , H. A. Henderson , and R. Allen. 2013. *Diet and Nutrition Survey of Infants and Young Children, 2011*. https://www.gov.uk/government/publications/diet-and-nutrition-survey-of-infants-and-young-children-2011.

[mcn70132-bib-0018] Mameli, C. , S. Mazzantini , and G. Zuccotti . 2016. “Nutrition in the First 1000 Days: The Origin of Childhood Obesity.” International Journal of Environmental Research and Public Health 13, no. 9: 838. 10.3390/ijerph13090838. 27563917 PMC5036671

[mcn70132-bib-0019] McCance, R. A. , and E. M. Widdowson . 2014. McCance and Widdowson's the Composition of Foods (7^th^ Edition. Royal Society of Chemistry).

[mcn70132-bib-0020] Nicholas, J. , L. Stevens , L. Briggs , and L. Wood . 2013. *Pre‐School Food Survey*.

[mcn70132-bib-0021] Nutritics . (2025). Research Edition (v6.14) [Computer Software]. Dublin. Retrieved From. www.nutritics.com.

[mcn70132-bib-0022] Office for Health Improvement and Disparities . 2025, 11 June. National Diet and Nutrition Survey 2019 to 2023. Office for Health Improvement and Disparities. https://www.gov.uk/government/statistics/national-diet-and-nutrition-survey-2019-to-2023.

[mcn70132-bib-0023] Ofsted . 2024. Check If Childcare Is Registered. Ofsted. https://reports.ofsted.gov.uk/childcare.

[mcn70132-bib-0024] Parker, M. , F. Lloyd‐Williams , G. Weston , J. Macklin , and K. McFadden . 2011. “Nursery Nutrition in Liverpool: An Exploration of Practice and Nutritional Analysis of Food Provided.” Public Health Nutrition 14, no. 10: 1867–1875. 10.1017/S1368980011000887. 21729488

[mcn70132-bib-0025] Pearce, J. , C. Harper , D. Haroun , L. Wood , and M. Nelson . 2011. “Short Communicationkey Differences Between School Lunches and Packed Lunches in Primary Schools in England in 2009.” Public Health Nutrition 14, no. 8: 1507–1510. 10.1017/S1368980010003605. 21272423

[mcn70132-bib-0026] Pearce, J. , and C. J. Wall . 2023. “School Lunch Portion Sizes Provided for Children Attending Early Years Settings Within Primary Schools: A Cross‐Sectional Study.” Journal of Human Nutrition and Dietetics 36, no. 5: 1887–1900. 10.1111/jhn.13183. 37278164

[mcn70132-bib-0027] Pearce, J. , and C. J. Wall . 2025. “Packed Lunch Provision and Consumption in Early Years Settings in Sheffield: A Cross‐Sectional Study.” Journal of Human Nutrition and Dietetics 38, no. 3: e70066. 10.1111/jhn.70066. 40369978 PMC12079081

[mcn70132-bib-0028] Scientific Advisory Committee on Nutrition . 2023. Feeding Young Children Aged 1 to 5 Years. TSO (The Stationery Office). https://assets.publishing.service.gov.uk/media/662a4a4d690acb1c0ba7e616/SACN-Feeding-young-children-aged-1-to-5-full-report-revised.pdf. 10.1017/S0029665126102250 41748544

[mcn70132-bib-0029] UK Government . 2024. Get Information About Schools. UK Government. https://www.get-information-schools.service.gov.uk/.

[mcn70132-bib-0030] Wall, C. J. , and J. Pearce . 2023. “Energy and Nutrient Content of School Lunches Provided for Children Attending School‐Based Nurseries: A Cross‐Sectional Study.” Public Health Nutrition 26, no. 12: 2641–2651. 10.1017/S1368980023002331. 37921199 PMC10755416

[mcn70132-bib-0031] Warren, E. , P. Boadu , J. Exley , L. Williams , B. Erens , and C. Knai . 2023. “Knowledge and Use of Voluntary Food and Drink Guidelines In English Nurseries? Results From a Nationally Representative Cross‐Sectional Study.” Food Policy 122: 102573. 10.1016/j.foodpol.2023.102573.

[mcn70132-bib-0032] World Health Organisation . 2016. Report of the Commission on Ending Childhood Obesity. World Health Organisation. https://www.who.int/publications/i/item/9789241510066.

[mcn70132-bib-0033] Yoong, S. L. , M. Lum , L. Wolfenden , et al. 2023. “Healthy Eating Interventions Delivered in Early Childhood Education and Care Settings for Improving the Diet of Children Aged Six Months to Six Years.” Cochrane Database of Systematic Reviews 6, no. 6: CD013862. 10.1002/14651858.CD013862.pub2. 37306513 PMC10259732

